# Wie unterstützen Leitungskräfte der Weiterbildung organisationsinterne digitale Veränderung? Eine qualitative Fallstudie im Kontext von Volkshochschulen

**DOI:** 10.1007/s40955-023-00243-z

**Published:** 2023-05-17

**Authors:** Johannes Wahl, Dörthe Herbrechter

**Affiliations:** 1 Eberhard-Karls-Universität Tübingen, Tübingen, Deutschland; 2 Ruprecht-Karls-Universität Heidelberg, Heidelberg, Deutschland

**Keywords:** Digitale Transformation, Führung, Organisationsentwicklung, Professionalisierung, Volkshochschulen, Digital transformation, Leadership, Organisational development, Professionalisation, Adult education centres

## Abstract

Führungskräfte nehmen im Rahmen der digitalen Transformation nicht zuletzt an der Schnittstelle zwischen Organisation und Umwelt eine wichtige Position ein. Der vorliegende Beitrag untersucht explorativ aus Sicht der Leitungskräfte, (1) welche Impulse und Akteure aus der organisationalen Umwelt als wichtig erachtet werden, (2) welche Maßnahmen und Aktivitäten ergriffen werden, um organisationsintern digitale Veränderungen anzustoßen und (3) welche Formen der Führung dazu genutzt werden. Die Datengrundlage basiert auf halbstandardisierten Leitfadeninterviews mit Leitungskräften an Volkshochschulen (*N* = 34), die inhaltsanalytisch induktiv und deduktiv ausgewertet wurden. Die Ergebnisse verdeutlichen die Komplexität der Anforderungsstruktur an das Führungshandeln und zeigen auf, welche Impulse die Führungskräfte für eine interne Veränderungsdynamik setzen können. Zudem weisen die Befunde auf ein Zusammenspiel verschiedener Formen der Führung hin, die im Kontext der Weiterbildung mit spezifischen Herausforderungen verbunden sind.

## Abstract (en)

Due to their opportunities to exert influence, leading staff members occupy a central position in the context of digital transformation, both within the organisation and at the interface between the organisation and the environment. Based on an explorative study with leading staff members of adult education centres, this paper examines, (1) which impulses and actors from the organisational environment are considered important, (2) which measures and activities are taken to initiate internal organisational changes and (3) which forms of leadership are used for this purpose. The data basis is formed by semi-standardised guided interviews with managers at adult education centres (*N* = 34), which were analysed inductively and deductively. The results illustrate the complexity of the demands on leadership and show which impulses the leading staff members can set for an internal dynamic of change. Furthermore, the findings point to an interplay of different forms of leadership that are associated with specific challenges in the context of adult education.

## Einleitung: Veränderungen der Umwelt als Anlass für digitale Veränderungsprozesse in Volkshochschulen

Wie alle Organisationen sind auch Weiterbildungsorganisationen darauf angewiesen, Veränderungen ihrer Umwelt wahrzunehmen, zu verarbeiten und auf sie zu reagieren (Dollhausen 2020; Herbrechter und Schrader, 2018; Nerdinger et al. 2019). Solche äußeren Veränderungen können sich etwa auf finanzielle oder rechtliche Rahmenbedingungen beziehen; sie können aber auch eine Folge disruptiver Krisen sein, wie etwa die Migrations- und Flüchtlingsbewegungen seit 2015, der Russland-Angriffskrieg oder die Covid-19-Pandemie. Für Weiterbildungsorganisationen bringen solche äußeren Veränderungen oftmals auch Veränderungserfordernisse im Inneren mit sich. So weisen Daten des nationalen Bildungsberichts beispielsweise darauf hin, dass sich Volkshochschulen vor allem in den ersten Jahren nach den Migrations- und Flüchtlingsbewegungen im Jahr 2015 besonders ausgeprägt im Bereich von Integrationskursen engagiert haben (Autor:innengruppe Bildungsberichterstattung 2022). Dies dürfte auch Folgen für ihre organisationsinternen Prozesse haben. Denn gemäß den Richtlinien des Bundesamts für Migration und Flüchtlinge (BAMF) geht die Zulassung als Kursträger für Integrationskurse mit spezifischen Anforderungen an die Qualifikationsprofile und Arbeitsumgebung für Lehrende einher (BAMF 2023), denen Volkshochschulen bei der Personalrekrutierung und der späteren Zusammenarbeit entsprechend Rechnung tragen müssen. Soll das Bildungsangebot auf eine digitale Planung, Organisation und Durchführung ausweichen, wie etwa während der Covid-19-Pandemie, erfordert dies zum einen ad hoc eine verbesserte technische Ausstattung und beschleunigte Digitalisierung aller internen Prozesse. Zum anderen werden die Formen der Zusammenarbeit und das professionelle Handeln grundlegend mit neuen Anforderungen konfrontiert (Schmidt-Hertha 2020; Christ et al. 2021).

Im Kontext der Weiterbildung treffen diese exemplarisch skizzierten Umweltveränderungen auf eine besondere Situation der Organisation. Anders als im schulischen Kontext ist für einen Großteil der Weiterbildungsorganisationen ein spezifisches institutionelles Arrangement prägend, das sich anhand der Bildungsberichterstattung kursorisch so beschreiben lässt: In der Weiterbildung ist ein relativ hoher Anteil des Personals selbständig auf Honorarbasis und oftmals in mehreren Organisationen parallel tätig. Unabhängig vom Beschäftigungsverhältnis arbeitet ein Großteil in Teilzeit. Während pädagogisch-planend-disponierende Aufgaben für gewöhnlich in Festanstellung erbracht werden, wird ein erheblicher Teil der Lehre auf Honorarbasis und nebenberuflich ausgebracht (Autorengruppe Bildungsberichterstattung 2016, S. 151–152, 154; Autorengruppe wb-personalmonitor 2016). Für Weiterbildungsorganisationen beinhaltet dieses spezifische Arrangement Vor- und Nachteile: Einerseits eröffnen ihnen die Honorarverträge und Teilzeitbeschäftigungen (auch bedingt durch die größere Zahl der beschäftigten „Köpfe“) ein hohes Maß an Flexibilität und Innovativität in der Angebotsplanung sowie geringe Kosten und Pflichten, vor allem für die Honorarkräfte (z. B. mit Blick auf Sozialversicherungsleistungen oder Kündigungsschutz; Jambon 2015). Andererseits sind mit diesen Beschäftigungsbedingungen auch spezifische organisationsinterne Herausforderungen verbunden, wie beispielsweise die Gewährleistung einer hinreichenden Interaktionsdichte zwischen den Beschäftigten, Kosten für zusätzliche Arbeitsplätze oder die Koordination der arbeitsteiligen Zusammenarbeit (Ulich 2011). Für die Gruppe der Lehrenden kommt hinzu, dass Honorarverträge kaum direkte Steuerungsmöglichkeiten und Weisungsbefugnisse eröffnen (Jambon 2015). So finden sich Leitungskräfte der Weiterbildung in der Situation wieder, dass ihre Möglichkeiten der gezielten Einflussnahme genau für diejenige Personalgruppe begrenzt sind, die der Organisation gegenüber den Teilnehmenden „ein Gesicht“ verleiht und die für die Erfüllung des Organisationszwecks von zentraler Bedeutung ist (Hahnrath und Herbrechter 2022).

Leitungskräfte der Weiterbildung sind bereits, bedingt durch diese vielfältigen, im Umfang und in der Absicherung variierenden Beschäftigungsbedingungen mit spezifischen Anforderungen konfrontiert, die sich angesichts der steigenden Ansprüche an die Effektivität und Effizienz der Weiterbildung sowie der eingangs beschriebenen gesellschaftlichen Veränderungen weiter ausdifferenziert haben dürften (Koschorreck und Gundermann 2021; Jenner 2022; Schrader 2019). Gerade in Zeiten, in denen äußere Veränderungen auch Veränderungen im Inneren der Organisation erforderlich machen (wie etwa die pandemiebedingt verstärkten Digitalisierungserfordernisse), kommt Leitungskräften eine besondere Verantwortung zu. Zusätzlich zu ihren regulären Führungsaufgaben gilt es, die notwendigen Veränderungen aktiv voranzutreiben, sie zu erklären, für sie zu motivieren, für Unterstützung und Beteiligung zu sorgen, gegebenenfalls erforderliche Kurskorrekturen vorzunehmen und Widerstände zu bearbeiten (Blessin und Wick 2017; Kraus 2020; für die Weiterbildung siehe auch Franz 2014). Wie Leitungskräfte der Weiterbildung dies unter den Bedingungen von Teilzeitarbeit als „Standard“ und einer großen Zahl neben- oder freiberuflich tätiger Lehrkräfte buchstäblich „managen“, ist bislang allerdings noch wenig erforscht.

Die digitalisierungsbezogene Weiterbildungsforschung hat in den vergangenen Jahren eine enorme wissenschaftliche Produktivität entwickelt, die sich beispielsweise an dem 2019 gegründeten „Netzwerk Erwachsenenpädagogische Digitalisierungsforschung“, Schwerpunktbefragungen des wb-monitors (z. B. Koscheck et al., 2022), Themenschwerpunkten wissenschaftlicher Fachzeitschriften oder an entsprechend ausgerichteten Monographien, Sammelbänden und Einzelbeiträgen (z. B. Bernhard-Skala et al. 2021; Breitschwerdt und Egetenmeyer 2022; Denninger und Käpplinger 2021; Engels und Egloffstein 2021; Robak et al. 2022; Rohs und Ganz 2016; Schmidt-Hertha et al. 2020) ablesen lässt. Im Fokus stehen dabei aber vor allem die Angebots- und die Lehr-Lernebene sowie damit verbundene technische und professionelle Voraussetzungen (Schmidt-Hertha 2020). Weniger im Fokus ist bislang demgegenüber die Ebene der Weiterbildungsorganisation, der Führung sowie der Umweltakteure und -impulse, die für den organisationsinternen Umgang mit Digitalisierungserfordernissen als relevant wahrgenommen werden.

An diese Forschungslage knüpft der vorliegende Beitrag an, indem er die Wahrnehmung und Verarbeitung von Digitalisierungsanforderungen auf der Ebene der Weiterbildungsorganisation aus Sicht der Leitungskräfte exploriert. Als Datenbasis dient die Studie „Digitale Transformation von Volkshochschulen“ (DiTra_VHS), in der Leitungskräfte zu zentralen Veränderungen in ihrer Einrichtung im Kontext der digitalen Transformation befragt wurden. Angesichts der skizzierten Forschungslage bearbeitet der vorliegende Beitrag die Teilfragestellung der Studie, wie Leitungskräfte als Schlüsselakteure für gelingenden Wandel digitale Veränderungen im Inneren der Organisation unterstützen. Dabei kann sich Unterstützung in vielfältiger Form realisieren, beispielsweise als Aufnahme umweltinduzierter Impulse, als Anregung und Umsetzung konkreter interner Veränderungen oder als veränderungsförderliche Gestaltung der Vorgesetzten-Mitarbeitenden-Beziehung. Vor diesem Hintergrund lässt sich die allgemeine Fragestellung des Beitrags in folgende Detailfragen differenzieren:

- Welche Impulse und Akteure der organisationalen Umwelt sind für die Initiierung digitaler Veränderungen in Volkshochschulen aus Sicht der Leitungskräfte relevant?
- Durch welche Maßnahmen und Aktivitäten befördern die befragten Leitungskräfte digitale Veränderungen im Inneren der Organisation?
- Wie unterstützen Leitungskräfte unter den besonderen Beschäftigungsbedingungen der Weiterbildung intern digitale Veränderungen durch Formen „ihrer“ Führung?

Mit diesen Fragen rückt der vorliegende Beitrag Führung als eine organisationale Rahmenbedingung des pädagogisch-professionellen Handelns angesichts zunehmender digitaler Veränderungsanforderungen in den Analysefokus. Mit Blick auf die Fallauswahl bezieht sich die vorliegende Studie auf der Ebene der Organisation ausschließlich auf Volkshochschulen. Dies zum einen, weil das Zusammenspiel aus Teilzeitbeschäftigung und hohem Anteil an neben- oder freiberuflicher Beschäftigung insbesondere für Volkshochschulen charakteristisch ist, so dass die zuvor beschriebenen weiterbildungsspezifischen Führungsanforderungen bei diesem Anbietertyp empirisch besonders gut aufsuchbar sind (Autorengruppe Bildungsberichterstattung 2016). Zum anderen ermöglicht die Auswahl von Volkshochschulen die Analyse digitaler Veränderungen in historisch etablierten, in ihrem Bestehen relativ stabilen Weiterbildungsorganisationen, die durch ihre vielfältigen, offenen Angebote den Weiterbildungsbereich in Deutschland entscheidend geprägt haben und nach wie vor prägen (Süssmuth und Eisfeld 2018). Um der spezifischen Führungssituation in Weiterbildungsorganisationen Rechnung zu tragen, bezieht sich das Sampling auf der Ebene der Leitungskräfte auf alle Personen, die für die Organisationsleitung (z. B. Gesamtleitung oder Geschäftsleitung) und/oder für die Leitung einer organisationsinternen Einheit (Fach- oder Programmbereichsleitung) verantwortlich sind. Alle interviewten Leitungskräfte sind zumindest teilweise mit der Führung von Teilzeitkräften konfrontiert; die Führung von freiberuflich Tätigen wird vor allem von den Fach- bzw. Programmbereichsleitungen übernommen.

Zur Beantwortung der Forschungsfragen werden zunächst organisationstheoretische Perspektiven entfaltet und die Forschungslage zur Digitalisierung bzw. digitalen Transformation im Feld der Weiterbildung skizziert (Kap. 2). Daran anknüpfend wird das Forschungsdesign der Studie DiTra-VHS beschrieben. Als Datengrundlage dienen halbstandardisierte Leitfadeninterviews mit insgesamt 34 Leitungskräften baden-württembergischer Volkshochschulen, die deduktiv und induktiv nach den Prinzipien der qualitativen Inhaltsanalyse nach Kuckartz und Rädiker (2022) ausgewertet worden sind (Kap. 3). Die Ergebnisdarstellung orientiert sich an den zuvor entwickelten Detailfragen. Dabei werden zunächst für organisationsinterne digitale Veränderungen relevante Impulse und Akteure der organisationalen Umwelt aus Sicht der befragten Leitungskräfte spezifiziert. In einem nächsten Schritt wird exploriert, durch welche Maßnahmen und Aktivitäten Leitungskräfte die digitale Transformation der Organisation befördern und schließlich wird dargelegt, wie die interviewten Leitungskräfte aus ihrer Sicht digitale Veränderungen durch von ihnen beschriebene Formen der Führung unter den spezifischen Beschäftigungsbedingungen der Weiterbildung unterstützen (Kap. 4). Auf Basis dieser Erkenntnisse erfolgt schließlich die Diskussion der empirischen Ergebnisse im Kontext der theoretischen Perspektiven und des Forschungsstandes (Kap. 5).

## Theoretischer Rahmen und Forschungsstand: Organisationale Veränderungen und Führung im Kontext der digitalen Transformation

### Organisationale Veränderungen und Führung

Aus organisationstheoretischer Perspektive sind organisationale Veränderungen selbstverständliche Ereignisse, auf die Organisationen in ihrem Funktionieren und Bestehen ebenso sehr angewiesen sind wie auf ein gewisses Maß an Stabilität (Kieser und Ebers 2019). Dabei wird oftmals unterstellt, dass sich Veränderungen vor allem an Organisation-Umweltschnittstellen vollziehen, während das Innere der Organisation stärker von Stabilität geprägt ist (Kühl und Muster 2016). Auch wenn sich organisationsinterne Strukturen, Routinen, Arbeits- und Kommunikationsformen durch eine höhere Persistenz auszeichnen mögen, unterliegen jedoch auch sie dem Wandel (Blessin und Wick 2017). Dieser kann sowohl organisationsintern durch Entscheidungs- oder Reifungsprozesse als auch organisationsextern z. B. durch Veränderungen der Marktbedingungen, des Ausbildungsniveaus, der demografischen Situation oder der im Rahmen des Beitrags interessierenden technologischen Entwicklung und deren Verfügbarkeit bedingt sein (Blessin und Wick 2017). Ganz gleich, auf welche Einflussfaktoren sich organisationale Veränderungen im Einzelnen je zurückführen lassen, den Führungskräften kommt hierbei eine besondere Bedeutung zu (Kraus 2020). Dies nicht zuletzt, da Führungskräfte aufgrund ihrer formalen Position über besondere Potenziale der Einflussnahme verfügen (z. B. Entscheidungs‑, Kontroll‑, Informations- und Sanktionsprivilegien; Etzioni 1975). In organisationalen Veränderungsprozessen übernehmen sie für gewöhnlich folgende Funktionen, die ein zielgerichtetes handelndes Zusammenwirken der Organisationsmitglieder auch in Zeiten des Wandels gewährleisten sollen:

„[…] den Wandel aktiv mittragen; den Grad der Einbeziehung der MitarbeiterInnen steuern; Interpretieren und motivieren: Deutungen nahelegen; Entscheidungen fällen und revidieren; mit Widerstand umgehen“ (Blessin und Wick 2017, S. 392).

Mal mehr, mal weniger explizit verweisen diese fünf verschiedenen Funktionen darauf, wie Führungskräfte durch ihr Verhalten auf ihre Mitarbeitenden Einfluss nehmen, um einen gelingenden Wandel wahrscheinlicher zu machen. So können sie die notwendigen Veränderungen top-down anweisen oder bottom-up, orientiert an den Impulsen der Mitarbeitenden, moderieren. Sie können die Partizipationsmöglichkeiten der Mitarbeitenden einschränken oder ausdehnen; Erreichtes stabilisierend oder als veränderungsbedürftig interpretieren, um Veränderungen zu legitimieren; Entscheidungen gemäß oder jenseits bewährter Programme treffen und Widerstände der Mitarbeitenden zeitweise übergehen oder aktiv bearbeiten (Blessin und Wick 2017).

Wie Führungskräfte durch ihr Verhalten in einem gewünschten Sinne auf Mitarbeitende einwirken, zählt zu den klassischen Fragen der Organisationsforschung, die seit den 1930er-Jahren im Zuge der Human Relations Bewegung mehr und mehr Beachtung gefunden hat und insbesondere in der psychologisch geprägten Führungsforschung zum Gegenstand empirischer Forschung gemacht wird (Weibler 2012). Inzwischen liegt eine Reihe unterschiedlicher Klassifikationsschemata vor, die verschiedene Grunddimensionen des Führungsverhaltens systematisieren, wobei sich die Schemata vor allem darin unterscheiden, wie viele und welche Dimensionen sie jeweils berücksichtigen (Weinert 2004; v. Rosenstiel und Nerdinger 2020). Als grundlegende, in zahlreichen empirischen Studien identifizierte Verhaltensdimensionen der Führung lassen sich folgende drei Dimensionen anführen (Weibler 2012):

- Aufgabenorientierung: Der Fokus liegt auf der Aufgabenerfüllung und dem Erreichen der Organisationsziele; die Führungskraft misst eindeutigen Aufgabendefinitionen, der Sicherung der Kooperation und Kommunikation zwischen den Mitarbeitenden, Vorschriften und Anregungen zur Aufgabenerfüllung eine hohe Bedeutung bei.
- Mitarbeitendenorientierung: Rücksichtnahme auf die persönlichen Interessen und Bedürfnisse der Mitarbeitenden; die Führungskraft trägt Sorge für das Wohlergehen der Mitarbeitenden und respektiert Vorschläge und Einwände.
- Entscheidungspartizipation: Beteiligungsmöglichkeiten der Mitarbeitenden an Entscheidungen. In Orientierung an eine Unterscheidung von Tannenbaum und Schmidt lassen sich die Möglichkeiten zur Entscheidungspartizipation idealtypisch auf einem Kontinuum in sieben Führungsstile differenzieren (autoritär, patriarchalisch, informierend, beratend, kooperativ, delegativ, autonom), wobei die Entscheidungsfreiräume von autoritär zu autonom für die Führungskraft geringer und für die Mitarbeitenden größer werden (Tannenbaum und Schmidt 1958; Wunderer 2009).

Auch wenn die Ergebnisse der Führungsverhaltensforschung in der modernen psychologischen Führungsforschung durchaus kritisch betrachtet werden (z. B. fehlende Berücksichtigung weiterer Einflussvariablen, primäre Erfassung auf der Basis subjektiver (Selbst‑)Einschätzungen, z. T. eingeschränkte Qualität der Messinstrumente), fanden und finden die drei angeführten Grunddimensionen des Führungsverhaltens auch noch in späteren führungspsychologischen Studien Berücksichtigung (z. B. Fiedlers Kontingenztheorie, Fiedler und Mai-Dalton 1995; normatives Führungsmodell nach Vroom und Yetton, Jago 1995; Reifegradmodell nach Hersey und Blanchard; Hersey und Blanchard 1977; v. Rosenstiel und Nerdinger 2020).

In der organisationsbezogenen Weiterbildungsforschung hat die Bedeutung des Führungsverhaltens für organisationale Veränderungsprozesse bislang nur wenig Berücksichtigung gefunden (Herbrechter 2018; Überblicksdarstellung zum Thema Führung und Führungsstil siehe z. B. Nuissl 1998; Dust 2012; für managementbezogene Befunde siehe z. B. Sauer-Schiffer 2000; Robak 2004; Uhmann 2011; für aktuelle Befunde der Berufsgruppenforschung zur weniger emotional geprägten Führung in Weiterbildungsorganisationen siehe Hodapp 2020). Für organisationale Veränderungsprozesse in öffentlich geförderten Weiterbildungsorganisationen konnte Kil (2003) zeigen, dass neben „Beanspruchung“ und „Verwaltung“ auch „Führung“ zu denjenigen Einflussfaktoren zählt, die sich nachteilig auf organisationalen Wandel auswirken können. Ebenfalls für den Bereich der öffentlich geförderten Weiterbildung weist die Studie von Walter (2002) darauf hin, dass die befragten Volkshochschulleitungen (*N* = 137) ihren eigenen Führungsstil überwiegend als kooperativ beschreiben und für sich selbst Weiterbildungsbedarfe mit Blick auf eine professionelle Ausübung der Führungsaufgabe wahrnehmen. Ein ähnlicher Befund zeigt sich auch in einer kontrastiven Fallstudie von Herbrechter (2018), die sich allerdings, wie die Studie von Walter, nicht auf organisationale Veränderungen bezieht (für ein idealtypisches Anforderungsprofil für Volkshochschulen; Feld 2007), sondern vielmehr Vorstellungen angemessener Führung von Leitungskräften und ihre institutionelle Rahmung untersucht. Für den Kontext der öffentlichen Weiterbildung weisen die Befunde darauf hin, dass eine starke Regelorientierung sowie Merkmale kooperativer Führung für das Führungsverständnis prägend sind (Herbrechter 2016a). Zugleich machen die Befunde darauf aufmerksam, dass sich Leitungskräfte der Weiterbildung kontextübergreifend von autoritärer Führung distanzieren; diese wird von ihnen als einseitige Machtausübung oder als Einforderung unbedingter Gefolgsbereitschaft eindeutig negativ konnotiert (Herbrechter 2016b).

Vor diesem Hintergrund nehmen wir als sensibilisierendes Konzept (Blumer 1954) für unsere eigene Datenanalyse an, dass die befragten Volkshochschulleitungskräfte mit Blick auf die Unterstützung digitaler Veränderungen durch Führung vor allem auf kooperative Formen des Führungsverhaltens Bezug nehmen. Dies entspricht nicht nur den bisherigen Befunden der organisationsbezogenen Weiterbildungsforschung. Vielmehr zeigen auch die Befunde der psychologischen Führungsforschung, dass kooperatives Führen insbesondere unter der Bedingung einer hochqualifizierten Mitarbeiterschaft – die im Weiterbildungsbereich mit knapp zwei Dritteln akademisch Beschäftigter gegeben zu sein scheint (Autor:innengruppe Bildungsbericht 2022) – von zentraler Bedeutung ist (v. Rosenstiel und Nerdinger 2020, S. 34). Da das Führungsphänomen in der Weiterbildung bislang noch wenig erforscht ist, beziehen wir uns für die deduktiven Basiscodierungen unseres Datenmaterials auf die zuvor beschriebenen grundlegenden Dimensionen des Führungsverhaltens als Heuristik für die Definition des deduktiven Codierleitfadens, die einerseits eine erste Strukturierung des Datenmaterials ermöglicht und andererseits aufgrund ihres allgemeinen, branchenübergreifenden Charakters eine weitere Spezifikation für den Weiterbildungsbereich im Zuge der induktiven Feincodierung erlaubt (Kuckartz und Rädiker 2022).

Da Volkshochschulen im Vergleich zu anderen Anbietern eine relativ große Zahl an neben- oder freiberuflich tätigen Lehrenden beschäftigen (Autorengruppe wb-personalmonitor 2016), nehmen wir zudem an, dass sich die Beschreibungen des Führungsverhaltens primär auf die festangestellten pädagogischen Mitarbeitenden und Verwaltungskräfte beziehen, da sie – anders als die Lehrenden – zu den Leitungskräften in einem direkten formalen Weisungszusammenhang stehen (Jambon 2015).

### Organisationale Veränderungen und digitale Transformation

Aus techniksoziologischer Sicht (Schrape 2021) lassen sich die organisationalen Veränderungen in der Weiterbildung als Phänomene der digitalen Transformation begreifen. Darunter wird ein soziotechnischer Rekonfigurationsprozess verstanden, der auf die „sukzessive Verfestigung neuartiger soziotechnischer Prozesszusammenhänge durch die soziale Aneignung digitaltechnischer (Infra‑)Strukturen und die damit verbundene Rekonfiguration gesellschaftlicher Ordnungsmuster“ (Schrape 2021, S. 87) ausgerichtet ist. Aus dieser Perspektive werden die Auswirkungen bzw. Aneignungsprozesse neuer Technologien in einem bestimmten gesellschaftlichen Sektor als sektorale Eingriffstiefe bezeichnet und daran anknüpfend verschiedene Varianten gradueller Transformation differenziert (Dolata 2011). Sie bestimmen das Ausmaß, in dem mit dem Auftauchen einer neuen Technologie strukturelle Veränderungen im jeweiligen Sektor einhergehen. Versteht man Weiterbildung als einen „heterogenen und nur lose gekoppelten, rechtlich kaum reglementierten Bildungsbereich“ (Schrader 2011, S. 97), kann sie als ein solcher Sektor gefasst werden. Diese Perspektive erlaubt es nicht nur, die konkreten Aneignungsweisen der digitalen Transformation auf verschiedenen Ebenen der Weiterbildung zu erforschen, sondern auch die damit verbundenen gesellschaftlichen Kontextualisierungen. Angesichts sich verändernder Abläufe im Bereich des Planungshandelns und der Verfügbarkeit digitaler Technologien zur Gestaltung inter- und intraorganisationaler Koordination rückt dabei auch der Wandel von Führung in den Fokus.

Phänomene der digitalen Transformation werden in der Weiterbildungsforschung in jüngerer Zeit auch mit Blick auf Veränderungen im Inneren der Organisation untersucht. So machen Ergebnisse der wbmonitor-Umfrage 2019 zum Thema Digitalisierung darauf aufmerksam, dass sich Weiterbildungsorganisationen jenseits der Lehr-Lernebene in der Verwendung digitaler Technik insbesondere auf das Bildungsmarketing via Website (64 % der Anbieter; *N* = 1551) oder Social Media (62 %) konzentrieren. Telearbeit/Home Office (43 %) und digitale Arbeitstreffen (35 %) spielen im Vergleich noch eine geringere Rolle (Christ et al. 2020), dies dürfte sich aber nicht zuletzt aufgrund der Covid-19-Pandemie künftig ändern. Die zunehmende Bedeutung von Aufgaben und Anforderungen der digitalen Transformation spiegelt sich auch in den von Weiterbildungsorganisationen ausgebrachten Stellenanzeigen wider. So konnten Alke und Uhl (2021) mithilfe einer längsschnittlichen Inhaltsanalyse von Volkshochschulstellenanzeigen (*N* = 332) zeigen, dass der Anteil an Stellenanzeigen mit Digitalisierungsbezug von 2016 bis 2020 zunächst moderat und von 2019 auf 2020 deutlicher angestiegen ist, wobei digitale Aufgaben überwiegend als Add-On für bereits etablierte Berufsprofile ausgeschrieben wurden.

Im Bereich des Medieneinsatzes verweisen die Ergebnisse von Breitschwerdt et al. (2022) mit Fokus auf die allgemeine und berufliche Weiterbildung darauf, dass die Verwendung von Computern bzw. Laptops (97 % der Mitarbeitenden, Mitwirkenden und Lehrenden; *N* = 111) auf der Lehr-Lernebene den Kern der digitalen Mediennutzung bildet. Auf dieser digitalen Infrastruktur aufbauend verwenden je 86 % der Studienteilnehmenden Anwendungssoftware und Videokonferenzanwendungen, während für didaktisch strukturierte digitale Medienangebote der Einsatz von Videos (81 %) gegenüber anderen Gestaltungsmöglichkeiten wie bspw. explorativer Lernumgebungen (31 %) oder Extended Reality-Anwendungen (14 %) dominiert (ebd.). Auf Basis der wbmonitor-Umfrage 2021 wird zudem deutlich, dass der Einsatz von Live-Onlinetrainings (von 3 % auf 51 %; *N* = 567 zu *N* = 628) und Lernplattformen (von 17 % auf 36 %) sowie Anwendungen im Kontext von Social Media (5 % auf 12 %) im Vergleich zwischen 2019 und 2021 auf der Lehr-Lernebene gestiegen ist (Koscheck et al. 2022). Dies korrespondiert mit den Ergebnissen des Adult Education Survey 2020, die ebenfalls auf die zunehmende Nutzung und Thematisierung von digitalen Medien auf der Lehr-Lernebene hinweisen (Bundesministerium für Bildung und Forschung 2022).

Mit Blick auf die organisationale Angebotsstruktur werden insbesondere für die letzten beiden Jahre deutliche Veränderungen erkennbar. Ergebnisse der wbmonitor-Umfrage 2020 verweisen darauf, dass im Vergleich zur vorpandemischen Situation (notgedrungen) eine stärkere Akzentuierung von Online- und Hybridangeboten erfolgte. Dabei zeigt sich, dass diese Anpassungsleistung je nach Anbietertyp in unterschiedlichem Ausmaß variiert und bspw. Anbieter wissenschaftlicher Weiterbildung ihr Angebot in der Tendenz umfassender als beispielsweise Volkshochschulen digitalisieren konnten (Christ et al. 2021). Widany et al. (2022) analysieren auf Basis von Leistungsdaten der Volkshochschul-Statistik und einer Sonderbefragung der Volkshochschulen Änderungen in der Angebotsstruktur während des ersten Lockdowns 2020 und arbeiten heraus, dass es nicht nur einen Einbruch der insgesamt realisierten Angebote gab, sondern die stattgefundenen Lehr-Lernsituationen eher als Einzelveranstaltungen denn als klassische Kurse gestaltet wurden. Zudem identifizieren sie besonders bei einer insgesamt durchschnittlich verringerten Angebotsvielfalt deutliche Ausfallquoten in den Programmbereichen Gesundheit und Kultur, während die Programmbereiche Sprachen, Integrationskurse und Qualifikationen für das Arbeitsleben in diesem Zeitraum sogar Zuwächse verzeichnen konnten. Koscheck et al. (2022) arbeiten mit Blick auf die Verbreitung von Onlineformaten in den verschiedenen Reproduktionskontexten der Weiterbildung (Schrader 2011) heraus, dass Universitäten, Fachhochschulen und wissenschaftliche Akademien in den Jahren 2020 und im ersten Halbjahr 2021 mit 58 % (2020; *N* = 1528) und 83 % (erstes Halbjahr 2021; *N* = 1382) Onlineveranstaltungen unter allen Angeboten am stärksten auf dieses Format setzten. Bei allen Anbietertypen ging dieser Zuwachs zulasten von Präsenzveranstaltungen, wobei vor allem die Volkshochschulen (−32 %) diese besonders reduzierten.

Mit diesen Veränderungen auf organisationaler Ebene gehen auch spezifische Anforderungen an die Organisationsmitglieder einher. Scheidig (2021) sowie Koschorreck und Gundermann (2021) identifizieren mit Blick auf das Handeln von Führungskräften eine komplexe Anforderungsstruktur, die je nach bereits existenten Organisationsstrukturen und Ausmaß der digitalen Transformation variieren kann. Mit Blick auf Führungsverhalten werden folgende Aspekte relevant:

- Kommunikation, Kollaboration und Dateimanagement in der Organisation mittels digitaler Infrastrukturen
- Kooperation mit anderen Organisationen zur Identifizierung von Synergieeffekten und Entwicklung neuer Angebote
- Rekrutierung und Professionalisierung des mikrodidaktischen Personals

Insgesamt lässt sich im Anschluss an Schmidt-Hertha (2020) nach wie vor feststellen, dass die bisherige Weiterbildungsforschung vor allem auf Angebotsveränderungen, die digitale Mediennutzung auf Angebotsebene und die damit verbundenen Anforderungen für Lehrende und Lernende in den Mittelpunkt rückt. Fragen der Organisation, der veränderten Zusammenarbeit und der Führung werden demgegenüber bislang weniger adressiert (Bernhard-Skala et al. 2021). Dies erscheint jedoch notwendig, um den Organisationen bzw. ihren Leitungskräften Erkenntnisse bereitzustellen, um ihre Einrichtung in einer dynamischen organisationalen Umwelt adäquat zu positionieren. Ein Forschungsprojekt, dessen Ergebnisse zur Bearbeitung dieses Desiderats beitragen kann, wird nachfolgend vorgestellt.

## Studiendesign: Das Projekt „Digitale Transformation von Volkshochschulen“

Die Veränderungen in Volkshochschulen im Zuge der digitalen Transformation bilden den Gegenstand des Projekts „Digitale Transformation von Volkshochschulen“ (DiTra_VHS), das die Forschungsfrage „Welche beruflichen und organisationalen Veränderungen verbinden Leitungskräfte der Erwachsenen‑/Weiterbildung mit der digitalen Transformation in ihrem Zuständigkeitsbereich?“ untersucht. Den Ausgangspunkt der Studie bilden die zusätzlichen Herausforderungen für professionelles pädagogisches Handeln in der Weiterbildung im Zuge der digitalen Transformation. Das Ziel des Projekts besteht darin, das Ausmaß der digitalen Transformation von Volkshochschulen zu erforschen. Dazu werden Bedingungen, Kontexte und Strategien der Professionalisierung und des organisationalen Wandels untersucht. Im Rahmen dieses Beitrags richtet sich das Analyseinteresse auf das Führungsphänomen als organisationale Rahmenbedingung des pädagogisch-professionellen Handelns mit Blick auf Bearbeitung digitaler Veränderungsanforderungen. Die selektierte Personalgruppe der Leitungskräfte ist für die Erforschung organisationaler Veränderungen besonders geeignet, da sie in der Organisation durch ihre herausgehobene Position und privilegierten Einflusspotenziale an der Schnittstelle zwischen Organisation und Umwelt eine Schlüsselfunktion in derartigen Prozessen einnimmt (Blessin und Wick 2017).

Als Methode der Datenerhebung wurde ein explorativer qualitativer Querschnitt mittels explorativer Experteninterviews mit Leitungskräften von Volkshochschulen in Baden-Württemberg zwischen November 2021 bis Mai 2022 gewählt. Zur Strukturierung der Interviews wurde ein halbstandardisierter Leitfaden mit sieben Themenkomplexen eingesetzt. Pandemiebedingt wurden bis auf ein Interview alle Gespräche mittels Videokonferenztool durchgeführt und aufgezeichnet. Das Sample umfasst 34 Interviews (28 Leitungen und sechs Fach- oder Programmbereichsleitungen) mit einer durchschnittlichen Dauer von 75:04 min. Die Transkription der Audiospur erfolgte in Anlehnung an Dresing und Pehl (2018). Für die Analyse des Datenmaterials wurde die inhaltlich-strukturierende qualitative Inhaltsanalyse nach Kuckartz und Rädiker (2022) angewandt.

Für den vorliegenden Beitrag wurden die 34 Interviews vor dem Hintergrund der o. g. drei Detailfragestellungen analysiert. Zur Erfassung relevanter Impulse und Akteure der organisationalen Umwelt sowie konkreter Maßnahmen und Aktivitäten der Leitungskräfte mit Blick auf die digitale Transformation der Organisation wurden angesichts der zuvor skizzierten Forschungslage die Daten induktiv zu Kategorien aggregiert. Anschließend wurden die Ergebnisse im Rahmen der eigenen Arbeitsgruppe kommunikativ validiert. Die Analyse der gewählten Formen der Führung orientiert sich an den theoretischen Überlegungen und empirischen Befunden der psychologischen Führungsverhaltensforschung. Hierfür wurden die in Kap. 2 skizzierten allgemeinen Grunddimensionen des Führungsverhaltens sowie das Führungsstilkontinuum nach Tannenbaum und Schmidt als allgemeine Heuristik zugrunde gelegt, innerhalb des deduktiven Codierleitfadens mit Definitionen und Codiervorschriften präzisiert und in einem nächsten Schritt induktiv spezifiziert.

## Empirische Ergebnisse

Die nachfolgende Ergebnisdarstellung gliedert sich in drei Bereiche. Im ersten Abschnitt werden die für Veränderungen im Kontext der digitalen Transformation bedeutsamen Impulse und Akteure der organisationalen Umwelt aus Sicht der Leitungskräfte empirisch spezifiziert (Abschn. 4.1). Anschließend wird exploriert, mit Hilfe welcher Maßnahmen und Aktivitäten die Leitungskräfte eine digitale Transformation der Organisation befördern (Abschn. 4.2) und schließlich wird dargelegt, wie die Leitungskräfte aus ihrer Sicht Veränderungen im Kontext der digitalen Transformation durch von ihnen beschriebene Formen der Führung unter den spezifischen Beschäftigungsbedingungen der Weiterbildung unterstützen (Abschn. 4.3).

### Rezeption von Impulsen und Erwartungen der organisationalen Umwelt

Als zentrale Voraussetzung für gelungenes Führungsverhalten im Kontext der digitalen Transformation kann die Rezeption der Impulse aus der institutionellen Umwelt angesehen werden. Veränderungen im Organisationsumfeld und Erwartungen organisationsexterner Akteure werden dabei als exogene Impulse zur Initialisierung organisationaler Veränderungen eingestuft, die nicht zuletzt durch entsprechende Entscheidungen der Leitungskräfte in die Organisation überführt und adaptiert werden können (Herbrechter 2018). Analog zu bisherigen Ergebnissen der Weiterbildungsforschung (z. B. Christ et al. 2021) wird die Covid-19-Pandemie als zentrale Umweltveränderung wahrgenommen, die sich auf die Gestaltung der Angebotsformate und die mikrodidaktische Einbindung digitaler Medien auswirkt:

„Und dann kam die Pandemie und der Verband hat gesagt macht S6. Das war bestimmt.“ (P26_93)

Wie das Beispiel zeigt, erfolgte in diesem Zeitraum nicht nur die bloße Nutzung eines trägerspezifischen Online-Netzwerks. Vielmehr verdeutlicht es die verpflichtende Ausrichtung an den Erwartungen eines organisationsexternen Akteurs. Als ebenso relevant werden die Erwartungen externer Datenschutzbeauftragter angesehen, wobei hier die Nichtnutzung spezifischer Software mit einem als unzureichend eingestuften Sicherheitsniveau begründet wird. Die Erwartungen einer weiteren Akteursgruppe erhalten bei der Gestaltung der Kursanmeldung Aufmerksamkeit:

„Auf der anderen Seite haben wir natürlich schon auch gerade im Onlinebereich was die, das Kursbuchen und so angeht, auch da haben wir einen hohen Druck von den Teilnehmern. Die sagen, hey, wieso kann ich nicht bei euch meinen Kurs online buchen?“ (P13_50)

Es zeigt sich, dass die Bedarfe der Adressatinnen und Adressaten eine wesentliche Orientierungsgröße für die Einrichtungsleitungen darstellen. Das Spektrum der Erwartungen reicht dabei vom Wunsch nach mehr digitalisierungsbezogenen Angeboten (P2_65), Onlineveranstaltungen zur Überbrückung von Lockdowns (P46_34) bis zu konkreten Austauschformaten, um sich im Hinblick auf Probleme der eigenen Mediennutzung beraten zu lassen (P39_54).

Eine weitere Form exogener Impulse umfasst Stimuli zur organisationsspezifischen Ausgestaltung der digitalen Transformation. Hierbei greifen die Leitungskräfte auf eine Vielzahl an Akteuren zurück, um den Anforderungen des Transformationsprozesses gerecht zu werden. Exemplarisch verdeutlicht dies das nachfolgende Zitat:

„Wir haben ja jetzt auch das/ Bundesland 3 hat ja das Förderprogramm 25 ausgegeben. Da haben wir uns auch/ Also das haben wir voll ausgeschöpft und haben da einfach diverse Tools, Hardware angeschafft, also Kamera oder Konferenzsystem und so weiter. Also da haben wir auch mit Unterstützung vom Volkshochschulverband 2, da haben wir gleich/ als es angeboten wurde, dass man da Unterstützung bekommt, kam dann auch ein Referent zu uns und hat dann mit uns die Räume/ haben wir gleich angeguckt. Wir haben auch eine externe IT-Betreuung. Der kam dann auch und dann haben wir geguckt, was macht Sinn bei uns.“ (P41_42)

**Figure 1:**
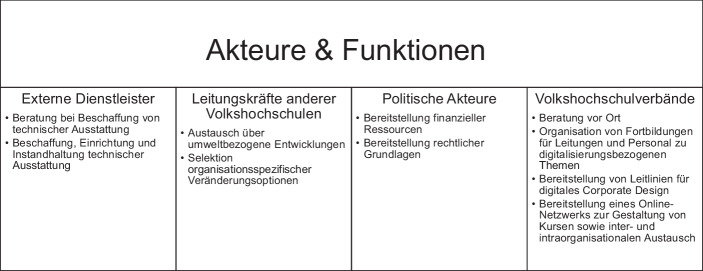
  Wie dieser Auszug exemplarisch belegt, nutzen die Leitungskräfte solche institutionalisierten Austauschformate und weitere informelle Gesprächskontexte, um aus der Fülle an Möglichkeiten adäquate Optionen für die eigene Organisation zu selektieren. Dabei wird es sowohl als hilfreich eingestuft, auf Organisationen mit vergleichbarer Größe zuzugehen (P44_104), als auch mit Volkshochschulleitungen ins Gespräch zu kommen, die im Bereich der digitalen Transformation als weiter fortgeschritten wahrgenommen werden (P26_101).
- *Politische Akteure* (bspw. Landesregierung oder lokale Entscheidungstragende) tragen zu organisationsinternen Veränderungsprozessen bei, indem sie (in)direkt finanzielle Ressourcen zur Verfügung stellen. Im vorliegenden Fall wird vor allem ein bundeslandspezifisches Förderprogramm für Volkshochschulen im Kontext der Digitalisierung erwähnt. Es ermöglicht den Organisationen den Aufbau bzw. Ausbau der digitalen technischen Ausstattung und stellt zudem Ressourcen bereit, um das Personal im Bereich digitalisierungsbezogener Themen zu qualifizieren. In ähnlicher Weise wird die digitale Transformation des Sektors durch die finanzielle Förderung von vier digitalen Pilot-Volkshochschulen unterstützt. Ziel ist hierbei im Sinne eines nicht näher definierten Best-Practice-Ansatzes, innovative Lern- und Organisationsszenarien im Bereich der digitalen Transformation und im Kontext der jeweiligen soziogeografischen Lage der Organisationen zu forcieren. Die befragten Leitungskräfte bestätigen in diesem Zusammenhang die Wirksamkeit der intendierten Unterstützung (P26_107). Darüber hinaus fungieren rechtliche Grundlagen im Bereich Datenschutz als Orientierungslinien beim (Nicht)Einsatz spezifischer Software bzw. digitaler Infrastrukturen (P1_146).
- *Volkshochschulverbände* auf Landes- und Bundesebene nehmen aus Sicht der Studienteilnehmenden eine besondere Stellung ein. Sie fungieren einerseits als Interessenvertretung der Einzeleinrichtungen gegenüber politischen Akteuren (P42_28) und stellen andererseits einen wichtigen Unterstützungskontext für die Organisationen dar. Dies dokumentiert sich anhand verschiedener Impulse, die sie im Rahmen der digitalen Transformation setzen. So bieten sie, wie das vorige Zitat zeigt, organisationsspezifische Beratung vor Ort an, führen Fortbildungen zu verschiedenen digitalisierungsbezogenen Themen für Leitungskräfte und Personal durch (P18_66, P47_58), unterstützen durch Leitlinien im Bereich des digitalen Corporate Design (P48_60) und stellen den Volkshochschulen zudem ein Online-Netzwerk für die Gestaltung von Kursen, aber auch zum inter- und intraorganisationalen Austausch zur Verfügung (P13_84).

Bei der Prozessierung der digitalen Transformation bzw. Schaffung begünstigender Rahmenbedingungen nehmen unterschiedliche Akteure je spezifische Funktionen ein. Durch den Kontakt zu ihnen erhalten die Leitungskräfte steuerungsrelevante Informationen, die in die organisationsinternen Führungs- bzw. Koordinationsprozesse einfließen (Abb. 1).^1^

- *Externe Dienstleister* werden von den Befragten herangezogen, um Beratung für eine technische Ausstattung zu erhalten, die an die Bedarfe der jeweiligen Organisation angepasst ist (P39_58). Zudem werden ihre Dienstleistungen in Anspruch genommen, um die organisationale technische Ausstattung zu beschaffen, einzurichten und instand zu halten (P16_72).
- *Leitungskräfte anderer Volkshochschulen* bilden eine weitere Grundlage für Impulse zur digitalen Transformation der eigenen Einrichtung. Der gegenseitige Austausch über umweltbezogene Entwicklungen und organisationale Veränderungsoptionen wird von den Befragten als zentrale Ressource wahrgenommen:
  „Und da sind wir dann auch als VHSen untereinander eine Quelle an Inspirationen und an Ideen. Dafür gibt es auch die Fachkonferenzen, die wir haben. Wo man dann zueinander kommt und sich auf einer vertrauensvollen Ebene gut austauscht.“ (P40_44)

Zusammenfassend lässt sich festhalten, dass Führung in Weiterbildungsorganisationen und hier insbesondere das Treffen strategischer Entscheidungen zu organisationalen Veränderungen durch die Rezeption der organisationalen Umwelt mitstrukturiert zu sein scheint. Die exogenen Impulse der Umwelt diffundieren keinesfalls zwangsläufig unmittelbar in die organisationalen Strukturen ein; vielmehr stellen sie Entscheidungsoptionen der Führung dar, die je spezifisch angeeignet und verarbeitet werden (z. B. Oliver 1991).

### Digitale Transformation: Maßnahmen und Aktivitäten der Leitungskräfte

Der Analysefokus des folgenden Teilkapitels richtet sich darauf, durch welche konkreten Maßnahmen und Aktivitäten Leitungskräfte die Prozessierung digitaler Veränderungen im Inneren der Organisation befördern.

#### Digitale Medienausstattung ausbauen

Die befragten Leitungskräfte verweisen in den Gesprächen durchgängig darauf, dass die Verfügbarkeit digitaler Endgeräte und Infrastrukturen eine essentielle Bedingung für die digitale Transformation der eigenen Organisation darstellt. Dies steht im Einklang mit den Erkenntnissen des Forschungsstandes zum Aufgabenzuwachs für Leitungskräfte im Kontext der digitalen Transformation (Scheidig 2021). Wie auch in den Studienergebnissen von Breitschwerdt et al. (2022) bereits anklingt, bilden stationäre und mobile Endgeräte die Basis der digitalen Medienausstattung. In den Daten zeigt sich die Präferenz, ausschließlich mobile Endgeräte anzuschaffen, um deren Einsatzmöglichkeiten orts- und personenvariabel zu gestalten (P13_34, P8_70) sowie Home Office zu ermöglichen (P26_81, P40_38). Geräte wie z. B. Dokumentenkameras, Kameras mit automatischer Gesichtsverfolgung oder Smartboards werden von den Leitungskräften demgegenüber je nach räumlichen Gegebenheiten und möglichen aktuellen wie zukünftigen Einsatzszenarien ausgewählt. Dabei spielt die (prospektive) Umsetzung von Hybridangeboten („Also wir nennen es immer Ausstattungspaket für einen vollwertigen Hybridunterricht“ (P16_76)) eine maßgebliche Rolle. Eine weitere zentrale Funktion nehmen cloudbasierte Infrastrukturen ein, die für die Flexibilisierung von internen Austauschprozessen genutzt werden (P26_16).

#### Individuelle Professionalisierung ermöglichen

Analog zu den Ergebnissen des wbmonitor 2019 (Christ et al. 2020) und des Adult Education Survey 2020 (Bundesministerium für Bildung und Forschung 2022) verweisen auch die befragten Leitungskräfte auf die hohe Bedeutung von Lernkontexten im Umgang mit digitalen Medien bzw. Infrastrukturen sowie den damit verbundenen Phänomenen der digitalen Transformation (z. B. Datenschutz) für das Personal. Dieser Bedarf resultiert aus Sicht der Leitungskräfte primär aus den Anforderungen der veränderten Medienausstattung und tangiert dabei sowohl den Bereich der mediendidaktischen Kompetenz als auch der medienbezogenen personalen Kompetenz (Rohs et al. 2017) des Personals. Diese Konstellation führt dazu, dass die Leitungskräfte Lernkontexte bereitstellen, die je nach Verfügbarkeit von finanziellen, personellen und zeitlichen Ressourcen durch sie selbst gestaltet oder aber von diesen organisiert werden.

Die Daten der DiTra_VHS-Studie stützen zudem die Erkenntnisse des wbmonitor 2021 zur Bedeutung von internen Weiterbildungsveranstaltungen: Auch während der pandemischen Situation bilden arbeitsplatznahe Schulungen den Schwerpunkt der Lernkontexte für das Personal (Koscheck et al. 2022). Für den untersuchten Kontext der öffentlich finanzierten Weiterbildung ist dabei auch maßgeblich, dass die Angebote nicht zwangsläufig nur für das festangestellte Personal zugänglich sind. Je nach Organisationsgröße und Ressourcenausstattung begegnen Leitungskräfte ihrem Personal auf verschiedenen Ebenen und in verschiedenen Rollen, was zu zusätzlichen Herausforderungen in der Beziehungsgestaltung führen kann. Neben gruppenförmigen Angeboten unterstützen die interviewten Leitungskräfte einzelne Mitarbeitende in coachingähnlichen Einzelkonstellationen, die nicht selten in zeitintensive Beratungssettings münden (P1_77).

Ergänzend zu diesen arbeitsplatznahen Möglichkeiten der individuellen Professionalisierung ermöglichen die Leitungskräfte dem Personal auch formale Lernkontexte z. B. in Form von Fortbildungen. Im Vergleich zu Formen des Coachings zeigt sich dabei jedoch ein entscheidender Nachteil:

„Wir haben natürlich Fortbildungsangebote vom Verband, vom VV2. Wir haben interne Fortbildungsangebote. Nur (.), es ist ein Zeitproblem, sage ich mal so: Ich erlebe das so, dass das oft nicht so einfach ist, den ganzen Tag weg zu sein.“ (P2_183)

Der Datenauszug verdeutlicht nicht nur die Pluralität der Anbieter, sondern verweist auch auf den Zeitaufwand als zentrale Komponente des individuellen Weiterbildungsaufwands (Müller und Wenzelmann 2018).

### Führungsformen, die Veränderungen unterstützen

Der folgende Abschnitt widmet sich der Frage, *wie* die interviewten Leitungskräfte eine organisationsinterne digitale Transformation durch „ihre“ Führung unter den spezifischen Beschäftigungsbedingungen der Weiterbildung unterstützen. Standen im vorherigen Teilkapitel ihre Maßnahmen und Aktivitäten („Was“-Ebene) im Fokus, geht es im Folgenden darum, die zugunsten der innerorganisationalen digitalen Veränderung eingesetzten Formen des Führens („Wie“-Ebene) näher zu spezifizieren.

In Orientierung an den zuvor skizzierten Befunden der psychologischen Führungsverhaltensforschung (vgl. Abschn. 2.1) lassen sich die thematisierten Formen der Führung entlang der drei allgemeinen, branchenübergreifenden Grunddimensionen der Aufgabenorientierung, Mitarbeitendenorientierung und Entscheidungspartizipation strukturieren.

Dabei weisen die codierten Segmente darauf hin, dass für die befragten Leitungskräfte eine *aufgabenorientierte Führung* von besonderer Relevanz zu sein scheint. Jedenfalls entfällt ein Großteil der Codierungen auf Segmente, in denen die Sachaufgabenerfüllung und daran orientierte Aktivitäten der Leitungskräfte in den Mittelpunkt gerückt werden. Um die internen Arbeitsabläufe, die Kommunikation und die Aufgabenerledigung zu gewährleisten und zu verbessern, tragen sie vor allem dafür Sorge, dass sowohl die festangestellten pädagogischen Mitarbeitenden und Verwaltungskräfte als auch die primär auf Honorarbasis beschäftigten und daher vertraglich nur lose an die Organisation gebundenen Lehrenden über die erforderlichen technischen Arbeitsmittel verfügen (insbesondere P1, P2, P8. P9, P12, P13, P14, P16, P17, P25, P26, P27, P39, P41, P42). Zudem geben die befragten Leitungskräfte auch selbst Anregungen zur angemessenen Anwendung digitaler Medien, indem sie Workshops durchführen, anleiten und beraten. Entgegen unseren Erwartungen beziehen sie sich mit Blick auf die Befähigung der Mitarbeitenden und die Bereitstellung technischer Mittel zur angemessenen Aufgabenerledigung allerdings nicht vorrangig auf die organisationsinternen Arbeitsprozesse und das festangestellte Personal, sondern vielmehr auf die Veranstaltungsräume, das Angebot und die Lehrenden.

„Also, ja klar, das Pädagogische hat sich stark verändert, klar. Das ist, was ich meinte. Wenn man eben einen Onlinekurs durchführt. Es gab dazu auch extra Schulungen von uns natürlich. Wie führt man einen Kurs per U11 zum Beispiel durch? Es wird jetzt wieder Schulungen geben, speziell zu der Hybridausstattung, wo wir dann gezielte Dozenten eben einweisen und denen zeigen, wie das geht. Und es wird pädagogische dazu geben, wie man dann einen Hybridunterricht eben generell am besten gestalten kann“. (P16_144)

In dieser wie auch in anderen Interviewpassagen liegt nahe, dass sich die befragten Leitungskräfte in der von ihnen überwiegend beschriebenen aufgabenorientierten Führung deshalb insbesondere auf die Lehrenden und das Bildungsangebot beziehen, weil sie auf der Ebene des Lehr-Lerngeschehens – sicherlich auch noch einmal forciert durch die Covid-19-Pandemie – die größten Veränderungen und Entwicklungsbedarfe identifizieren. Dabei rekurrieren sie mit Blick auf die Befähigung der Lehrenden zum einen auf die Förderung ihrer grundlegenden Medienkompetenz (kompetente, kritisch-reflexive Verwendung digitaler Medien) und zum anderen auf die gezielte (Weiter‑)Entwicklung ihrer mediendidaktischen Kompetenz (didaktisch angemessene Verwendung digitaler Medien, um eine gelingende Vermittlung und Aneignung wahrscheinlicher zu machen) (Rohs et al. 2017). Mit Blick auf ihre Pflicht, für die Befähigung und individuelle Professionalisierung der Mitarbeitenden Sorge zu tragen, thematisieren die befragten Leitungskräfte in mehreren Passagen, dass sie zwar vielfältige non-formale und informelle Formen der Weiterbildung anregen, hierbei allerdings für die Gruppe der Lehrenden auch an Grenzen stoßen (P13, P2, P9_40). Denn aufgrund ihrer überwiegenden Beschäftigung auf Honorarbasis stehen die Lehrenden formal gesehen in keinem direkten hierarchischen Verweisungszusammenhang der Organisation (Jambon 2015), so dass sie zur Partizipation an berufsbegleitenden Formen der Weiterbildung nicht verpflichtet, sondern allenfalls durch niedrigschwellige Angebote oder monetäre Anreize aktiv motiviert werden können. Dass insbesondere Volkshochschulen dazu bereit sind, auch für die Weiterbildung der lehrenden Honorarkräfte – ungeachtet ihrer möglichen Mehrfachbeschäftigung bei verschiedenen Weiterbildungsanbietern – einzutreten, ist auch in älteren Daten des wbmonitor aus dem Jahr 2008 erkennbar. Auch hier zeigt sich, dass Volkshochschulen die Weiterbildung „ihrer“ Lehrenden z. B. durch interne Fortbildungsangebote, Vorträge oder die (anteilige) Kostenübernahme für externe Seminare (Ambos und Egetenmeyer 2009) aktiv unterstützen. Dieser Befund zeigt sich auch in den jüngeren Daten der DiTra-VHS-Studie: die interviewten Leitungskräfte beschreiben ein ähnlich ausgeprägtes Engagement, das von persönlicher Unterweisung bis hin zur Finanzierung von Fortbildungsgebühren für neben- oder freiberufliche Lehrende reicht (P17_72).

Dass die befragten Leitungskräfte Wert darauf legen, zu ihren festangestellten pädagogischen und administrativen Mitarbeitenden ebenso wie zu den lehrenden Honorarkräften eine gute soziale Beziehung zu pflegen und auf ihre Interessen, Bedürfnisse, Ängste und Belastungsempfindungen gleichermaßen einzugehen, spiegelt sich in den Codierungen zur *mitarbeitendenorientierten Führung* wider (vor allem P8, P9, P12, P14, P17, P25, P27, P40, P41, P42). Diese beziehen sich sowohl auf das pädagogische und administrative Personal als auch auf die Lehrenden, wobei die Anzahl der codierten Segmente für die Lehrenden deutlich geringer ausfällt. Obgleich die Leitungskräfte sich in vielfältiger Weise für die digitale Transformation „ihrer“ Organisation einzusetzen scheinen, machen sie auch wiederkehrend deutlich, dass digitale Veränderungen der Organisation limitiert sind. Als wichtigster begrenzender Faktor wird auf die Kapazitätsgrenzen des aktuell verfügbaren Personals verwiesen. In diesem Zusammenhang wird deutlich, dass sowohl die Wahrnehmung formaler Lernkontexte zum Aufbau relevanter Kompetenzen für bisherige Zuständigkeiten (P25_62) als auch weitergehende Impulse z. B. zur Nutzung neuer Marketingkanäle und die damit verbundene Übernahme neuer Zuständigkeiten zu Belastungsspitzen aufseiten des Personals führen, die es zu berücksichtigen und auszugleichen gilt.

Mit Blick auf die *Partizipationsmöglichkeiten* an organisationsinternen Entscheidungen zeigt sich schließlich (insbesondere P1, P2, P7, P8, P9, P12, P13, P14, P16, P17, P18, P25, P26, P27, P39, P40, P41), dass sich die Leitungskräfte in ihren Erzählungen erkennbar häufiger auf Entscheidungssituationen beziehen, in denen sie ihren Mitarbeitenden Partizipationsmöglichkeiten einräumen. Dabei beziehen sie sich insbesondere auf kooperative Formen der Entscheidungsfindung, die es den Mitarbeitenden ermöglichen, früh eigene Ideen und Lösungsvorschläge einzubringen und auf Veränderungsbedarfe aktiv hinzuweisen. Diese Mitwirkungsmöglichkeiten werden primär dem festangestellten Personal in solchen Situationen eröffnet, in denen es um die konkrete Umsetzung der organisationsinternen digitalen Transformation (z. B. in der Planung und Administration von Integrationskursen; Vorschläge für die Angebotsstruktur und didaktisch-methodische Variationen auf der Lehr-Lernebene, die Medienausstattung, zukünftige Marketingkanäle oder die Digitalisierung von Arbeitsroutinen) geht. Auf autoritäre, allein getroffene Entscheidungen scheinen die Leitungskräfte demgegenüber seltener und insbesondere in solchen Situationen zurückzugreifen, in denen ein schnelles Entscheiden erforderlich ist (z. B. mit Blick auf die Covid-19-Pandemie) oder in denen sie grundlegende Impulse anstoßen und die digitale Transformation der Organisation strukturell forcieren (z. B. Fokussierung auf ein spezifisches Tool; Funktionsrolle der/s Digitalisierungsbeauftragen).

## Diskussion

Der Beitrag rückte Führung als eine organisationale Rahmenbedingung für pädagogisch-professionelles Handeln unter den Vorzeichen der digitalen Transformation von Volkshochschulen in den Mittelpunkt des Interesses. Dabei richtete sich der Analysefokus darauf, empirisch zu spezifizieren, welche Impulse und Akteure der organisationalen Umwelt für die Initiierung digitaler Veränderungen aus Sicht der Leitungskräfte relevant sind. Zudem wurde untersucht, durch welche Maßnahmen und Aktivitäten Leitungskräfte den organisationsinternen digitalen Wandel bearbeiten und wie Leitungskräfte interne Veränderungen im Kontext der digitalen Transformation durch von ihnen beschriebene Formen der Führung unterstützen.

Die Ergebnisse zeigen die komplexe Anforderungsstruktur des Führungshandelns im Kontext der digitalen Transformation. Mit Blick auf die erste Fragestellung lässt sich festhalten, dass die Befragten einerseits mit einer Vielzahl an Akteuren und deren Erwartungen umgehen müssen, diese aber andererseits auch als Impulse für das eigene Führungsverhalten im Kontext der digitalen Transformation fungieren können. Diese Konstellation verdeutlicht die transmissiven Gestaltungsmöglichkeiten von Führungskräften bei der Selektion exogener Impulse. Eine besondere Rolle spielen hierbei auch Kooperations- und Konkurrenzkonstellationen. Die betreffenden Akteure können vor dem Hintergrund eines territorialbezogenen Bildungsauftrags durch digitale Strukturen ihre Angebote räumlich und zeitlich entgrenzen, was sowohl Potentiale für neue Angebote und Kooperationen als auch für Konflikte bietet. Beides zu untersuchen, ist Gegenstand weiterführender Analysen der DiTra-VHS-Daten. Hinsichtlich der zweiten Frage, *was* die Leitungskräfte zugunsten der organisationsinternen Digitalisierung tun, zeigt sich ein größeres Spektrum unterschiedlicher Maßnahmen zur Umsetzung der organisationsinternen Digitalisierung. Auch wenn das Verhältnis in den verschiedenen Fallorganisationen unterschiedlich akzentuiert sein mag, verlassen sich alle befragten Leitungskräfte nicht allein auf den Ausbau der digitalen Medienausstattung. Vielmehr koppeln sie die Förderung der digitalen Infrastruktur mit der Förderung der digitalen Kompetenzen des Personals. Dabei scheinen sie sich ganz besonders für die informelle Professionalitätsentwicklung zu engagieren, indem sie verschiedene Formate unterstützen, selbst anleitend aktiv werden und individuell coachen. Ergänzend schaffen sie zudem Anreize für (non‑)formale Formate beispielsweise durch Finanzierungsangebote für Fortbildungsmaßnahmen.

Mit Blick darauf, *wie* die befragten Leitungskräfte die organisationsinterne digitale Transformation durch Formen „ihrer“ Führung unterstützen (Frage 3) lässt sich zusammenfassend festhalten, dass sich die Führungsverhaltensbeschreibungen der befragten Leitungskräfte erschöpfend durch die aus der führungspsychologischen Forschung abgeleiteten drei Grunddimensionen des Führungsverhaltens strukturieren lassen. Die befragten Leitungskräfte nehmen auf alle drei Grunddimensionen Bezug. Anders als von uns angesichts des Forschungsstandes angenommen, beziehen sich die Leitungskräfte allerdings nicht primär auf Formen der kooperativen oder mitarbeitendenorientierten Führung. Vielmehr rekurrieren sie überwiegend auf Elemente einer aufgabenorientierten Führung, die primär auf die Erfüllung der Organisations- bzw. Arbeitsgruppenziele angelegt sind. Inwiefern die befragten Leitungskräfte aufgabenorientierte Formen der Führung gegenüber einer mitarbeitendenorientierten Führung präferieren, lässt sich anhand des vorliegenden Datenmaterials nur schwer abschätzen. Aus Perspektive der psychologischen Führungsforschung lässt sich aber annehmen, dass sie sowohl aufgabenorientiert als auch mitarbeitenden- und partizipationsorientiert agieren und die jeweilige Ausprägung stark durch die Wahrnehmung der Situation beeinflusst ist (Blessin und Wick 2017). Die entsprechenden Passagen des Datenmaterials legen jedenfalls nahe, dass die interviewten Leitungskräfte aufgabenorientierte Formen der Führung in ihren Erzählungen nicht per se, sondern situationsbedingt und daher etwas stärker adressieren, weil sie die digitale Transformation ‚ihrer‘ Organisation zum Interviewzeitpunkt als unabgeschlossenen „Change“-Prozess wahrnehmen, den es zugunsten der Erfüllung der Organisationsziele und des damit verbundenen Bildungsauftrags weiter voranzutreiben gilt.

Dabei beschränken sie sich nicht allein auf das Personal bzw. die Mitglieder der Organisation im engeren Sinne. Vielmehr versuchen die befragten Leitungskräfte auch auf die lehrenden Honorarkräfte zugunsten einer professionellen Aufgabenerfüllung gezielt Einfluss zu nehmen – ein Befund, der sich arbeitsrechtlich nicht zwangsläufig nahelegt. Denn die Honorarbeschäftigung verleiht den Leitungskräften keine formale Weisungsbefugnis gegenüber den Lehrenden (Jambon 2015) und die im Zuge von Fortbildungsmaßnahmen erworbenen Kompetenzen können angesichts der häufigen Mehrfachbeschäftigung der Lehrenden auch anderen Anbietern en passant zugutekommen. Befunde der organisationsbezogenen Weiterbildungsforschung weisen allerdings darauf hin, dass insbesondere Volkshochschulen dazu bereit sind, ungeachtet ihrer vertraglich losen Kopplung an die Organisation, auch in die individuelle Professionalisierung der Lehrenden zu investieren (z. B. Ambos und Egetenmeyer 2009). Dies spiegelt sich auch in den Daten der DiTra_VHS-Studie wider. Zugleich machen sie aber auch auf spezifische Herausforderungen der digitalen Transformation unter den für die Weiterbildung typischen Beschäftigungsbedingungen aufmerksam. So kommt die Einbindung der Lehrenden dann an Grenzen, wenn es um die Partizipation an Entscheidungsgremien und Mitwirkungsmöglichkeiten geht. In diesen Situationen scheinen die Leitungskräfte auf die Innovativität der Lehrenden eher zu verzichten und die Organisationsgrenze aktiv herzustellen, indem sie Möglichkeiten der Entscheidungspartizipation primär ihren festangestellten Mitarbeitenden einräumen. Und auch mit Blick auf die Einbindung der festangestellten Mitarbeitenden stoßen sie insofern an Grenzen, als die Umsetzung bottom up entwickelter Veränderungsimpulse, vermutlich auch bedingt durch das verbreitete Teilzeitarbeitsmodell, herausfordernd ist und die zusätzliche Übernahme neuer Aufgaben (wie z. B. die Nutzung neuer Marketingkanäle) durch Kapazitäts- bzw. Belastungsgrenzen erschwert wird. Diese Ergebnisse liefern erste, induktiv ermittelte Ansatzpunkte für die weitere Spezifikation der drei Grunddimensionen des Führungsverhaltens für den Kontext der Weiterbildung in Form von Führung von Teilzeitkräften sowie von neben- oder freiberuflich Tätigen, die in der bisherigen organisationsbezogenen Weiterbildungsforschung bislang noch wenig Berücksichtigung gefunden haben.

Auch wenn die Ergebnisse instruktive Einsichten in die Veränderungsimpulse gewähren, die die Leitungskräfte innerhalb ihrer Einrichtungen setzen, sind sie in ihrer Reichweite limitiert. Die DiTra_VHS-Studie wurde explorativ als Querschnittserhebung umgesetzt, sodass eine verallgemeinernde Theoretisierung der Erkenntnisse nicht ohne Berücksichtigung des konkreten Erhebungskontextes möglich ist. Dabei ist zudem zu beachten, dass die Phänomene der digitalen Transformation nur anhand eines Anbietertyps erforscht wurden und dies mit Fokus auf ein Bundesland. Einschränkungen entstehen zudem aufgrund der nur impliziten Behandlung der Führungsthematik im Erhebungsinstrument, sodass nicht alle deduktiv möglichen Facetten des Führungsverhaltens und seine spezifische Ausformung unter den Beschäftigungsbedingungen der Weiterbildung in den Blick gerieten.

Ungeachtet dieser Einschränkungen zeigen die Ergebnisse deutliches Potenzial für weiterführende Forschung: Die transmissive Gestaltungsmöglichkeiten ließen sich etwa mittels Netzwerkanalysen des jeweiligen Organisation-Umweltverhältnisses im Kontext organisationaler Veränderungen näher analysieren. Hierbei wäre auch von Interesse, inwiefern die digitale Transformation Re-Konfigurationen zwischen externen und organisationsinternen Akteuren erforderlich macht und bisherige Routinen der Programmplanung verändert. Mit Blick auf die Führungsthematik ließe sich der innerhalb des Beitrags identifizierte Mix aus primär aufgaben-, aber auch mitarbeitenden- und partizipationsorientierter Führung näher spezifizieren, indem differenzierter untersucht wird, inwiefern Leitungskräfte auf unterschiedliche Akteurkonstellationen im Inneren der Organisation in gleicher oder unterschiedlicher Weise Einfluss nehmen, wie sie mit Widerständen gegen die digitale Transformation umgehen und wie sie die zuvor identifizierten, in den verschiedenen Segmenten des Weiterbildungsbereichs vermutlich unterschiedlich ausgeprägten Herausforderungen der Honorarbeschäftigung, des Teilzeitarbeitsmodells und der Kapazitätsgrenzen bearbeiten.

## Footnotes

1 Die Akteure in der nachfolgenden Aufzählung werden in alphabetischer Reihenfolge aufgelistet, ohne damit Priorisierungen hinsichtlich ihrer Bedeutung für die Studienteilnehmenden vorzunehmen. Die dabei genannten Funktionen entsprechen denen, die die Leitungskräfte expliziert haben.
